# “It is always me against the Norwegian system.” barriers and facilitators in accessing and using dementia care by minority ethnic groups in Norway: a qualitative study

**DOI:** 10.1186/s12913-020-05801-6

**Published:** 2020-10-15

**Authors:** Elżbieta Anna Czapka, Mette Sagbakken

**Affiliations:** Department of Nursing and Health Promotion, Faculty of Health Sciences, Oslo Metropolitan University, Pilestredet 32, 0130 Oslo, Norway

**Keywords:** Minority ethnic groups, Dementia care, Barriers and facilitators

## Abstract

**Background:**

Dementia is one of the greatest health challenges in the contemporary world. Due to several barriers to accessing health care services, elderly immigrants constitute a group that poses special challenges in dementia diagnosis and treatment. The aim of this study was to explore the barriers and facilitators in accessing and using dementia care services by minority ethnic groups in Norway.

**Methods:**

The study utilised a qualitative design. The participants included family caregivers of individuals from minority ethnic groups living with dementia, representatives of immigrant communities, and representatives of health and care personnel working with people living with dementia. Individual semi-structured in-depth interviews were carried out in 2018 and 2019 in Oslo and Akershus. Interviews were analysed using thematic analyses.

**Results:**

Several barriers and facilitators in accessing and using dementia care services were identified, the most important of which were related to lack of knowledge of dementia, lack of awareness of dementia care services, lack of language skills, culturally based differences, the organisation of Norwegian dementia care services, and immigrants’ socio-economic status. According to the study participants, having health care personnel in the family and further adaptation of dementia services to the needs of people with different cultural and linguistic backgrounds facilitate access to dementia services.

**Conclusions:**

The study shows the need to develop inclusive policies that promote a patient-centred approach to ensure that individuals from minority ethnic groups receive appropriate dementia care.

## Background

According to the United Nations (UN), the phenomenon of population ageing is a global trend [1].. The number of people aged 65 or over is expected to double from 703 million in 2019 to 1.5 billion in 2050 [2]. This is the result of declining fertility, increasing life expectancy, and migration of young people from developing to developed countries [3].

One of the dilemmas of global population ageing is the growing demand for health and social care [3]. Currently, the leading cause of death are not acute and infectious diseases but chronic and degenerative diseases [4]. They are linked to increasing dependency, long-term care needs and disability [3]. One such disease is dementia, considered by the World Health Organization (WHO) to be one of the greatest public health challenges of this century [5]. Dementia is a set of symptoms characterised by the deterioration of memory and other cognitive functions. It affects behaviour and the ability to perform everyday activities [6]. According to estimates, the prevalence of dementia worldwide almost doubles every 20 years (2015: 47.47 million, 2030: 82 million, 2050: 152 million) [6]. One of the main reasons for the increasing incidence of dementia is the rising rate of life expectancy [7]. Alzheimer’s disease (AD) accounts for 60–70% of dementia cases [5]. Most dementia diseases are progressive, but the speed at which the disease develops is individual [8]. According to a review of the studies on the benefits and challenges of timely diagnosis of Alzheimer’s disease, there are many potential benefits of early diagnosis of AD, such as better management of symptoms, implementation of coordinated care plans and postponement of institutionalisation [9]. Early diagnosis of dementia is difficult for many reasons. It is challenging to differentiate between the symptoms of early dementia and the normal ageing process [10]. Furthermore, diagnostic criteria for dementia are changing and remain controversial [11, 12]. A systematic review of the literature on prevalence and determinants of undetected dementia in the community and/or residential/nursing care show that more than 60% of dementia cases go undetected [13].

International research has shown ethnic differences in the use of dementia care services [14, 15], and delays in access to diagnostic services among older immigrants [16–18]. Studies have also found low rates of anti-dementia medication prescription and use [19–21] and a reduced likelihood of entering a long-term-care facility [19, 21]. Thus, it seems like elderly immigrants constitute a group that experience special challenges in dementia diagnosis and treatment [22–24].. A thematic synthesis on older immigrants’ access to health care shows that their experiences of accessing health care are influenced by factors such as health literacy, language barriers and differences in health care beliefs [25]. As a result, their use of health care services differs from that of natives [23, 26]. According to Ruspini’s overview of trends, policies and practices related to older immigrants in Europe, the under-usage of services by minority ethnic elderly is a common European trait [26]. A conceptual review of the effects of barriers to timely dementia diagnosis experienced by elderly from minority ethnic groups in the United States shows that older people from minority ethnic groups were more cognitively impaired than non-Hispanic whites and sought care at a later stage [27]. A meta-synthesis of qualitative studies shows that older immigrants experience several barriers to accessing dementia care [28]. The authors discussed the barriers with reference to two concepts: inadequacies and cultural habitus^1^/experience. Inadequacies refer to barriers in the form of deficits in education and service provision, such as a lack of information about dementia and health care systems within immigrant communities, and poor accessibility to such services. The concept of cultural habitus was used to describe the barriers related to cultural issues, such as community expectations regarding family care obligations and stigmatisation of dementia [28]. The review shows that only a few studies focused on facilitators in using dementia care by minority ethnic groups. Most of the facilitators were related to service-level interventions and included issues such as adapting services to patients’ needs, educating patients about dementia and improving screening [28].

Immigrants and Norwegian-born with immigrant parents constitute 17.7% of the total population of Norway. The five biggest immigrant groups are Poles, Lithuanians, Somalis, Swedes and Pakistanis. Since Norway’s history of immigration is relatively short, its immigrant population is much younger than the general population. In 2019, only 4% of immigrants and as much as 16% of the general population were 67 years old or older [29]. It is estimated that the number of older immigrants will increase from around 30,000 in 2013 to 300,000 in 2050 [30]. Until recently, little was known about the prevalence of dementia in immigrant groups in Norway because it was not possible to link registers containing data on medical diagnoses and ethnic backgrounds [20]. A national registry study showed that a significantly smaller proportion of immigrants was diagnosed with dementia or memory impairment. According to the authors, this may indicate a lower prevalence of milder forms of dementia among immigrants. However, the authors also pointed out other possible explanations of the study results, such as the existing barriers to accessing dementia care for people from minority ethnic groups or lower utilisation of primary health care services [20]. A recent study on health professionals’ experiences of identifying and diagnosing dementia among immigrants in Norway shows that general practitioners experienced many difficulties in assessing dementia in immigrant patients [31]. These were mainly due to language barriers and to health professionals’ lack of knowledge regarding appropriate diagnostic tools for patients with another cultural and/or linguistic background. Another study, conducted by the same authors, explored the views and experiences of family caregivers and professional caregivers regarding dementia care services in Norway [12]. The results show the complexity of the picture and the interplay of different factors. The authors argued that immigrants’ expectations and practices originate not only in cultural values, traditions and language-related barriers, but also in changes in gender roles and socio-economic status caused by migration processes.

Norway’s Dementia Plan 2020 emphasises the need to provide elderly immigrants with care that is adapted to their needs [32]. There are still many gaps in our knowledge on immigrants’ access to dementia care services in Nordic countries [33]. The present article is part of a larger study on understandings and experiences of and responses to age-related cognitive impairment among families from minority ethnic groups living in Norway. The main aim of this part of the study is to identify barriers and facilitators in accessing and using dementia care experienced by minority ethnic groups in Norway.

## Methods

### Study setting and participants

The study was conducted in 2018 and 2019 in Oslo and Akershus because this county has the highest density of immigrant groups in Norway. Data were collected over one year. In order to develop a comprehensive understanding of the phenomena, we used multiple data sources. The results presented in this paper are based on interviews with participants from three different groups: 1) individuals from minority ethnic groups with family members who a) have been diagnosed with dementia or b) had no diagnosis but experienced memory loss; 2) key representatives of immigrant communities; and 3) representatives of health and care personnel working with dementia patients. The first group of participants was recruited by contacting dementia coordinators, nursing homes, a memory clinic, day centres and the researchers’ personal networks. Initially, the researchers planned to include families from three major minority ethnic groups: Somalis, Pakistanis and Turks. However, due to difficulties in gaining access to participants, it was decided to use a convenience sampling method and include families from all minority ethnic groups where someone was living with age-related dementia/cognitive impairment, whether diagnosed or not. In five cases, the family caregivers declined to be interviewed. One of the potential participants did not want to specify the reason for declining, while two others believed that dementia was a family matter and should therefore not be discussed with outsiders. The representatives of the remaining two families explained that they did not have time to meet the researchers.

Eight families from minority ethnic groups were interviewed. The participants came from Somalia, Poland, Croatia, Pakistan, India, Turkey and one of the islands in the Atlantic Ocean. The participants’ ethnicity will not be presented in combination with other characteristics such as age, education, profession and other indirect identifiers in order to protect their identity. The family members living with dementia were present during four interviews. Three of them actively participated in the interview. One of them was able to give informed consent and the next of kin of two other participants’ relatives gave informed consent.

In order to gain more insight into immigrants’ perceptions of roles and responsibilities in elderly care and into how elderly immigrants and their families perceive and experience the Norwegian health system, five key representatives of immigrant communities (Polish, Turkish, Somali, Pakistani) were interviewed. Expert sampling was used, meaning that the researchers selected participants who knew both their own culture and the Norwegian culture. These participants were characterised by a high level of social involvement in the life of their own ethnic group and in Norwegian society, and were recruited through already established networks. In addition, six representatives of health and care personnel were interviewed. Expert sampling was used to recruit employees who had experience in working with patients with dementia. They were recruited through health care institutions and personal networks, and worked in nursing homes, a memory clinic and in home care service.

### Individual semi-structured in-depth interviews

Three different interview guides (Additional files 1, 2, 3) were used to explore the views, beliefs and experiences of all the participant categories. The interview guides were developed for this study, and included questions related to understandings of dementia in minority ethnic groups, seeking of formal care, and experiences of care provided by health services.

The interviews took place at a time and place convenient for the participants. The families were interviewed in their homes, except for one participant who wanted to meet the researcher in a library. The interviews lasted between 45 min and two and a half hours. Before each interview, most of the participants served coffee and snacks while the researcher asked introductory, warm-up questions. Each interview had its difficult moments when participants became highly emotional when talking about events from the past, such as when the relative was placed in a nursing home or was diagnosed with dementia.

The representatives of immigrant groups and health and care personnel were interviewed in their workplaces, homes or at the researcher’s office. The interviews lasted from 30 min to two hours. They were conducted in Polish, English, Norwegian, Somali or Punjabi. An interpreter was used during the interviews with Somali families. The interview with a Pakistani family caregiver was conducted by a research assistant who spoke Punjabi. The first author (EC) was present during the interview. EC speak Polish, English and Norwegian and conducted the interviews performed in these languages.

During all the interviews, the researcher discreetly took notes, describing various elements that were potentially important for data analysis, such as participants crying or showing visible signs of nervousness. After each interview, the recordings were immediately saved on the Service for Sensitive Data platform (TSD) and deleted from the voice recorder. The interviews were regularly transcribed and translated directly into English by the first author and the research assistant speaking Punjabi.

All the participants, including three individuals diagnosed with dementia, gave written informed consent to participate in the study. They were informed they could withdraw their participation without giving any reason.

### Reflexivity and positionality

The first author (EC) was aware of the importance of self-reflexivity, and examined her positionality and how it could influence the entire research process and its outcomes. During the interviews, EC continually negotiated her “insider/outsider” position. She experienced being perceived by the interviewees from minority ethnic groups to belong to “us” on account of her own minority ethnic background (Polish). At the same time, she experienced being perceived to belong to “them” on account of her position as a researcher and a representative of a Norwegian institution. The participants often seemed to believe that EC, having an immigrant background, shared their opinions about the Norwegian health care system and Norwegian culture. For example, one participant said “*You know how it is here, those doctors have no time for patients*”, and “*They eat bread all the time here, not like in our countries*”. Many Polish participants did not provide information about Polish culture or the health care system. They assumed that EC already had that knowledge due to her Polish background, and it was often necessary to ask additional questions to obtain the information needed to contextualise the participants’ experiences.

Due to the sensitive nature of the subject of the interviews, the first author’s aim was to provide a supportive environment so that participants felt comfortable to share their experiences [34]. The participants were treated as experts in order to minimise their perception of the first author as someone having a privileged position during the interview.

### Analysis

We used thematic analysis to identify, analyse, and report patterns within the data [35]. The interview transcripts were coded by EC for barriers and facilitators in accessing and using dementia care services. During the analysis we followed six phases described by Braun and Clarke [35]. In phase 1 we familiarised ourselves with the data by reading the interview transcripts and the notes from the interviews three times. In phase 2 the initial codes were generated by EC. Phase 3 comprised of searching for the general themes based on the generated codes by EC. In phase 4 the themes were reviewed and refined by both authors. We then named and defined the final themes and identified subthemes within each theme. For example, we identified three subthemes related to “lack of information”: lack of information about dementia, lack of information about available dementia care services, and misinformation about nursing homes in Norway. In phase 6 the content of the themes and subthemes was merged into generalised descriptions. The analysis was not a linear process, as we often moved back and forth between the different phases.

## Results

### Barriers

Even though our participants were ethnically diverse, we found common patterns during the data analysis. Four themes related to the barriers to accessing and using dementia care services by minority ethnic groups in Norway were identified: lack of knowledge about dementia, lack of awareness of dementia care services, culture-related barriers and barriers related to the Norwegian health services.

#### Lack of knowledge

Most of the study participants pointed to a lack of or limited knowledge about dementia as one of the key factors hindering minority ethnic groups’ access to dementia care services. An auxiliary nurse working in a nursing home explained why some people from minority ethnic groups may not seek help:

*I think that people often don’t know that their relatives suffer from dementia. They can’t read the symptoms. Perhaps they don’t know about illnesses such as dementia.*

Health care personnel provided many examples of patients and their families seeking help relatively late due to the conviction that memory loss is part of the normal ageing process. Some of the interviewed family caregivers confirmed that lack of knowledge about dementia and misconceptions about its symptoms and causes made them wait until it became very clear that something was wrong. For example, one family caregiver believed that her husband’s memory problems resulted from a very stressful event he had gone through.

The Somali participant, a young woman whose mother was living with dementia, described how long it took for the family to realise that the mother was ill:

*I would say it might be that we were too slow in getting help for her and that it took a while for us to understand that she had dementia. I think it would have been different if we had had more knowledge. It was after she burnt something at home that we decided that was enough and that we had to find out what was wrong.*

Other participants reported similar examples of delays in seeking help. The female participant whose husband had memory problems but who had not yet been diagnosed said they would contact their GP if “something really serious happens, for example, if he forgets to turn off the cooker”. She preferred to wait because it was “normal to forget when one turned fifty”. However, she worried a lot because she could see that her husband’s memory was deteriorating every day. He forgot appointments or what he was supposed to buy in the shop, and sometimes he did not engage in conversations.

One common finding from the interviews with health care personnel and representatives of immigrant communities was the opinion that ethnic origin and culture were overestimated as explanatory factors for access to dementia care. One of the interviewed representatives of health care personnel explained:

*I like to say that, fundamentally, people are very much alike. They have the same diseases, the same symptoms, but they look at things differently. This also applies for Norwegians with different backgrounds. Someone who has very little education, who has never worked with any intellectual work, is very different from someone who is a professor in biology. It has lots to do with a general background.*

The participant believed that socio-economic status, defined by the combination of education, income and occupation, is a more important factor revealing inequities in access to dementia care than minority ethnic background. Other participants representing minority ethnic groups confirmed the significance of educational background for immigrants’ understanding of dementia and of their health-seeking behaviour. A well-educated female participant from a minority ethnic group said:

*It is the lack of knowledge that makes people do that [hide dementia]. They don’t have much education. They should understand that this is not something to be ashamed of. It can happen to anyone.*

According to this participant, a lack of knowledge about dementia should be perceived not as part of a culture but rather as the result of poor education among many social groups.

### Lack of awareness of and misinformation about dementia care services

Lack of information about the available services was another barrier mentioned by the interviewed families and the representatives of immigrant communities. A woman who had a mother living with AD explained how difficult it was for immigrants to find information about what help is available:

*Immigrants know very little about how to help an elderly person with dementia in Norway. I must tell you that I have lived here for 22 years and there is a problem with it, because we don’t know where to get information and help.*

Other participants mentioned that it was not easy to navigate the Norwegian health care system. Consequently, many immigrants do not know who to contact first or what kind of help is available.

The findings also illustrate how a lack of reliable information can give rise to rumours and create distrust of Norwegian care institutions. It is normal practice in Norway to discontinue life-prolonging treatment when a patient is terminally ill. Since artificially administered fluid and nutrition are defined as life-prolonging treatment, these may be also discontinued. The procedure is applied to maintain the patient’s comfort, and each situation should be evaluated individually [36]. One of the participants, a representative of one of the immigrant associations, perceived the situation differently:

*Here, if patients can’t eat or can’t ask for food, they are left to die. In our country, we try to save lives at all costs. Here, there would be pressures on the family to decide about disconnecting the patient from a life support machine.*

She believed that the procedure dated back to a time when whole families would participate in killing elderly family members by throwing them off a cliff. Another participant, an auxiliary nurse working with people with dementia, presented a different explanation for discontinuing nutrition support. She believed that “it is probably too expensive for the state to keep the elderly alive”. One of the participants, a nurse working in a nursing home, explained that lack of information about the rationale behind discontinuing life-prolonging treatment can generate fear among families from minority ethnic groups and may discourage them from placing their elderly family members in care institutions.

Thus, our findings show that even if immigrants possess knowledge about dementia, they may not necessarily know where to seek help or may distrust Norwegian health care institutions due to misinformation.

#### Culture-related barriers

Our findings show that many barriers to accessing and using dementia care are related to immigrants’ cultural background, religion and internalised social and moral norms. Dementia seems to be a taboo subject in many cultures, and the study revealed examples of families trying to hide it. A medical doctor working with dementia patients provided many examples of families who did not want to talk about dementia with their friends, relatives or their GP because it would bring shame on the whole family. He explained that it was not that long ago since dementia was associated with shame even in Scandinavia, until increasing public awareness broke the taboo.

Religious beliefs was another important factor that hindered access to dementia services for some of the study participants. The two key representatives of the Somali community explained why people chose not to talk about dementia or to seek help:

*I think the majority would think that is a punishment from God because of something that they might have done.*

*I asked people and they think it is from God. Maybe not as a punishment but as forgiveness. Somali people are mostly religious people so when someone is diagnosed with a deadly disease, they would think that it is fate, and that God chose him because he loves him.*

Those two seemingly contradictory explanations refer to the religious origin of Somali people’s beliefs about the causes of dementia. Independent of which of the explanations representing the rationale, there is no reason to seek help since dementia is sent from God.

Another factor that could hinder access to dementia care was related to family obligations to provide care for the elderly. Our findings show that the willingness to fulfil family obligations was often associated with a lack of, or limited acceptance of, nursing homes and home-based services. The interviewed doctor said that his patients’ family caregivers often said things like: “*It [caregiving] is not a burden to me. My parents are not a burden to me*”. According to him, many relatives did not want to consider placing their parents in a nursing home. The family caregivers who were interviewed tended to refer to the norm of reciprocity, which requires children to care for their elderly parents. One of them, a young man, explained:

*That’s how it is in our culture. It is not forced. It is love I got from him when I was small. Now I am returning what I got. It is my responsibility. He has only me.*

One of the nurses working in the nursing home elaborated on the obligation to provide care for the elderly in families from minority ethnic groups by referring to her Pakistani neighbours:

*It can be a kind of a problem with mentality. I have Pakistani neighbours and they live together. All of them in one house. I think that the problem is not related to the Norwegian health care system. The problem is that they all care for each other.*

This quote illustrates the difference between two cultures of caregiving and associated caregiving regimes: the Pakistani familialistic caregiving model and the Norwegian model based on the state provision of care. The participant defined it as a “problem”, a barrier to immigrants’ use of dementia care services in Norway.

Our findings confirm that caring for the elderly constitutes a strong moral obligation in many cultures. Moral norms were often found to be deeply internalised, and any violation could invoke internal and external sanctions. The interviewed family caregivers, who grew up in their countries of origin, declared their willingness to care for family members living with dementia. However, we found that second-generation immigrants, who had internalised the moral and social norms of their parents’ culture and of the Norwegian culture, experienced many dilemmas related to care provision for their parents. One of the participants explained how people from her community would react if she placed her mother in an institution:

*People often talk about it in terms of shame: ‘Look what they have done to their parents’,* etc. *You can always hear something negative about it. If I put my mum in a day centre or other institution, I don’t think people would look at it positively. They would see that ‘the daughter cannot handle her mother, she is not good enough’. That is how it is in our community.*

The participant elaborated on different attitudes among the younger generation of immigrants who were brought up in Norway, attended Norwegian schools and worked in Norwegian institutions. According to her, second-generation migrants often do not perceive caring for the elderly as exclusively a women’s domain, even though women would assume the role of caregiver in their parents’ countries of origin. Also, those who represented the second generation seemed more open towards institutional care and solutions combining family care with institutional care. One of the participants described a situation she thought represented her generation:

*We feel obliged to care for our elderly. I do, however, think they [Turkish people] would like less responsibility if it was possible. If there was less social control and if the health authorities facilitated it better. I do believe they are somehow forced to help.*

This quote illustrates the dilemmas of the middle generation of minority ethnic groups. They have to operate between two caregiving regimes: familialistic regimes that characterise their parents’ countries of origin and the Norwegian caregiving regime characterised by defamilialisation through public provision of care.

#### Barriers related to the Norwegian health care system

All three groups of participants were concerned about the role of GPs in diagnosing dementia in patients from minority ethnic groups. One of their biggest concerns was the GPs’ lack of competence, the lack of time to diagnose dementia in specific patient groups, and a reluctance to refer patients to a specialist if necessary. The interviewed health care personnel talked about lack of culturally validated tests that make the process of diagnosing patients from minority ethnic groups very difficult. Many of the interviewed family members talked about difficulties in convincing their GPs that their relatives had memory problems and behaved differently. One of the participants said: “It took a year to convince the doctors that something was wrong”. Some participants felt ignored by their GP, as described by a family caregiver to a man in his fifties living with AD:

*Another matter is, when you feel sick no matter how many times you go to the GP and say that you are sick, your illness will not be recognised … They do a lot of tests and say ‘We will call you back if we find something’. Then you wouldn’t be contacted even if you had a disease. It may be because the treatment is expensive and the country doesn’t want to spend so much money on us with immigrant backgrounds..*

The participant further explained that after many visits to the GP with no results or feedback, they became discouraged and stopped going to the clinic. He was convinced that they were not treated seriously due to their minority ethnic background. Even though his cousin’s memory was deteriorating, he did not know how to convince the doctor to extend the diagnostics. He described how he felt helpless and started to distrust the Norwegian health care system. The cousin was diagnosed in one of the memory clinics a few years later, when his memory loss escalated and caused him problems at work.

The lack of linguistically competent services was considered to be one of the main barrier to accessing and using dementia care services within the Norwegian health care system. A doctor who diagnoses patients with memory problems talked about the challenges in diagnosing patients from minority ethnic groups due to language problems and to having to use interpreters, who are not trained to work with people suffering from cognitive impairments. A nurse with a minority ethnic background, working in a nursing home, paid special attention to language-related problems in her institution. She said that personnel often do not understand patients from minority ethnic groups, especially those living with cognitive impairments, and that as a result, nurses with the relevant language skills were asked to interpret. She also told how cases occur where no one speaks the patient’s language and, for various reasons, the personnel are unable to arrange for an interpreter. The participant had a feeling that there was an “unwritten rule” not to use interpreters at her institution, to avoid additional costs. She claimed this indirectly prevented personnel from booking interpreters, even in situations where doctors were examining a patient and it was considered necessary in order to provide the patient with proper care:

*When I meet them [senior staff at the nursing home] in the morning, all they talk about is how little money they have. The effect is that I don’t dare book an interpreter. Many colleagues are afraid of booking interpreters due to the economic situation.*

She further explained that there were situations where the lack of an interpreter made communicating with patients almost impossible and compromised the safety of both patients and health care personnel. For example, she was often asked to interpret conversations between health care personnel and patients and their families. However, she did not feel competent enough to translate medical terms:

*My colleagues tend to think that since I am already there I can interpret myself, but I tell them that I cannot interpret medical terms in my language. For instance, I am not able to translate ‘anticoagulants’ into my language, or ‘diuretic or blood pressure medication’. I don’t know how to translate such words.*

Due to difficulties in translating medical terminology, it was a very stressful experience for the participant. However, she was aware that a nursing home could not book interpreters every time health care personnel talked to patients from minority ethnic groups.

The interviewed families experienced language as the main barrier to using care services such as nursing homes, day centres and home-based services. It was at the root of many conflicts between families and service providers, as it created many misunderstandings regarding the institutional care. In addition, the families worried about their relatives in situations where the lack of language skills made effective communication impossible.

A young woman, a representative of an immigrant community, talked about the limited access to information about dementia and to health care services for those who do not speak Norwegian:

*People often lack sufficient information. You get everything in Norwegian. When you go to doctor, everything is in Norwegian. When you go to other places, everything is in Norwegian.*

She explained that elderly immigrants are rarely proficient in Norwegian, and that even those who are often lose this skill as their cognitive functions deteriorate, and they become totally dependent on family members.

Another barrier in the health care system that was often mentioned was that Norwegian nursing homes were “not prepared for immigrants”, and many were particularly critical about the food. One of the nurses from a minority ethnic group explained why Norwegian food can constitute a problem for older immigrants:

*I don’t like the food they make here. I can tell you, it is not good. All those sandwiches every day. Why do they eat so much bread? I would miss my food. I wouldn’t be able to stay here (in a nursing home). Norwegian food is very healthy, very healthy. It is healthy to eat cooked fish and carrot for dinner, but you must have been brought up on it. Otherwise, you don’t like it.*

The quote illustrates how much individual dietary habits and preferences depend on the culture of origin. The participant had knowledge of the health benefits of parts of the Norwegian diet, but she did not like it, and felt sorry for patients from minority ethnic groups living in nursing homes. Our findings show that the participants considered food to be a very important aspect of care. One family caregiver explained how people from home-based services showed a lack of cultural sensitivity by serving bread to his father:

*Do you know what happened when they came home to us? They brought him bread. In my culture, bread is not food. Maybe for breakfast. But still, it is not proper food. Proper dialogue would be good. They just say ‘these are the rules in Norway’.*

The participant defined serving bread to his father for lunch as a lack of respect. In his opinion, the Norwegian care system is not inclusive because immigrants are expected to adapt to Norwegian habits and rules, regardless of their needs. Another important factor was the possibility to practice one’s religion. Some of the participants said they would never place relatives with dementia in a Norwegian care institution because of the limited possibility to perform religious practices. One of the interviewed nurses explained that a priest came to the nursing home only once a week and some patients wanted to attend a service more frequently.

Health care personnel provided different examples of why nursing homes are not prepared for patients from minority ethnic groups. Besides the language barriers, staff shortages in nursing homes were frequently mentioned. This arose from dementia patients from minority ethnic groups needing more attention due to increasing communication problems.

Another barrier mentioned by some of the interviewed families of people living with dementia was the lack of or poor cooperation with people representing dementia care services. A man whose father had dementia reported difficulties in communicating with the dementia coordinator:

*People from the municipality, they speak Norwegian to my father. I told them that I know my father because I am with him every day. I know that he is losing his Norwegian language skills. When I ask him in our language ‘Where do you want to live?’, I get the same answer every day. If I ask him in Norwegian, the answer can change from hour to hour. He doesn’t understand the meaning. The people from the municipality are so stubborn.*

This quote illustrates a situation where the family caregiver tried to share his experiences regarding his father’s language skills with the dementia coordinator but felt ignored. After many misunderstandings and conflicts with municipal employees, he decided to stop using the care services and to care for his father himself. Other participants reported similar experiences of feeling disregarded by health care personnel. One family caregiver did not understand why the home care nurses refused help offered by the family caregivers during washing and dressing the patient. He said:

*The reason why we want to help is because of the difference in culture, language and religion. They can’t understand him as well as we do.*

The participant also expressed that he understood that the home care nurses were acting according to existing rules and regulations. However, in his opinion, it was important to consider the patient and his needs before all the regulations as “it would improve the patient’s mental, emotional and physical health”. The health care personnel, on the other hand, reported challenges related to minority ethnic families’ lack of understanding of how the care services and institutions are organised. One of the interviewed nurses, who worked in a nursing home, told us that she often had to negotiate between the immigrant families’ expectations, patients’ needs and organisational rules. She gave an example of the large extended family who wanted to accompany the older relative when she was dying. Our participant tried to find out whether her patient felt comfortable in a small room full of people. She said “It was impossible to breathe in that room”. When she understood that the dying woman was exhausted, she kindly asked the family to leave the room.

According to health care personnel, many immigrant families would choose to care for their relatives at home if they could get financial support from the state. One of the participants explained that care allowance is not automatically granted:

*There is something called omsorgslønn [care allowance] but it is very low pay. The municipality pays for this care allowance. The municipality always wants people to use the services from the municipality first. If that is not enough, then they say ‘We can pay a little bit of care allowance’.*

The narratives of the interviewed families confirmed the wish for more flexibility regarding help provided by the state. One of the family caregivers preferred to provide care for her mother living with AD at home. As it was exhausting, she wanted to admit her mother to a nursing home for a few days from time to time. However, she found it too expensive. Some of the other families wanted to get more frequent help from home-based services in order to continue providing care at home.

### Facilitators

We identified three factors facilitating access to and use of dementia care services for minority ethnic groups: having representatives of the medical profession in the family or among friends, adapting dementia care services to people’s language proficiency, and provision of reliable and comprehensive information.

#### Having a doctor in the family or from the same ethnic background

Two of the interviewed families pointed out the importance of the fact that their children, medical doctors, facilitated their access to dementia care services. The participant whose husband was living with AD said:

*My husband didn’t remember anything. He was asking vague questions like ‘Where am I?’ We immediately took him to the doctor in [name of the hospital]. My daughter is also a doctor and she arranged everything for us there. It was great that we didn’t have to worry about it.*

The participant further explained that she would not have known what to do if the daughter had not helped her. She did not know who to contact and, in addition, she was stressed because of her husband’s condition.

The other participant, a woman with early-stage dementia, was rather reluctant to see the doctor, although her family noticed that she was having memory problems. She said that she believed it was temporary and that she did not need medical intervention. However, her daughter, a medical doctor, convinced her to have a check-up and arranged a visit to her GP. The daughter described to the doctor the changes she observed in her mother’s behaviour. The mother, our study participant, described what happened during the consultation:

*We have had the same GP for 30 years. He told me: ‘I also forget, it’s not a big problem. I don’t think you need to take it further’. My daughter insisted, and he said he would send me to a specialist.*

The GP did not recognise early-stage dementia, but the daughter, who knew her mother very well and had medical training, was able to see the subtle changes in her mother’s behaviour. Had it not been for her daughter’s determination, the participant’s dementia diagnosis would have been postponed.

The other participant, whose mother was living with AD, described how a psychologist with the same ethnic background helped her navigate the dementia care services in Norway:

*I was feeling helpless. I didn’t know where to find help for my mum. And one day I went to a geriatric clinic in [name of a district in Oslo] and met a [nationality] woman who worked there. She was a psychologist. She helped us a lot. If I hadn’t met that woman, I don’t know what would have happened.*

The participant explained that the psychologist knew the differences between the [nationality] and the Norwegian health care systems and was aware of the barriers that [nationality] immigrants might experience in accessing dementia care services. She taught the participant how to navigate the Norwegian health services for people with cognitive impairment.

#### Adapting dementia care services to people’s language skills

Most of the study participants believed that health care institutions should be, at least to some extent, adapted to the needs of people from minority ethnic groups and that health care personnel should be more accommodating, especially in terms of language.

The participants gave examples of nursing homes where personnel have different ethnic backgrounds and speak different languages. According to many of the participants, dementia care services should be offered in the patient’s language. The representative of the migrant community said that nursing homes “*should set up duty rosters so that staff who speak the patient’s language is always available”*. Otherwise, according to other participants, easy access to an interpreter should be guaranteed in situations where good communication is crucial in order to ensure patients’ safety.

The findings provided a few examples of creative health and care personnel working at nursing homes and health institutions. These doctors, carers and nurses were open to collaborating with the families and to finding solutions that worked best. For example, one of the participants, whose mother has AD, initially refused any help offered by the home care services because of the language barriers:

*I wanted someone who spoke my mum’s language. Otherwise, it was very difficult to help my mum to shower, take her medicines or eat her meals. She doesn’t understand Norwegian.*

The participant moved her mother to Norway when it gradually became impossible for the mother to live alone. After some time, together with the home nurse services, the participant developed a system that enabled the nurses and care workers to communicate with her mother. For example, she told the mother that if someone knocked on the door three times, it meant that the driver from the day centre was coming to fetch her and she needed to go downstairs.

#### Provision of information

Many of the participants stressed the importance of providing both health care personnel and patients with information regarding dementia and patients’ rights. One of the family caregivers talked about a doctor who was very patient and who answered all his questions. The participant did not know much about dementia services in Norway and, in addition, he didn’t know where to find the required information.

One of the young nurses working in a nursing home stressed how important it was for health care personnel to be aware of the rights of patients from different cultural, social and religious backgrounds, and suggested that nursing students should receive the necessary information and skills:

*More focus on these [cultural diversity] challenges during training would have made it easier for us to perform our duties. We need to learn about patients’ rights. After reading about it, I realised that they have more rights than I thought. Ritual washing, special diets. Many don’t know about this.*

The participant also thought that immigrant patients should be actively informed about their rights.

The effective provision of information through different channels is another factor that, according to some of the participants, would facilitate immigrants’ access to dementia care services. The representative of one of the minority ethnic groups suggested that the authorities should actively inform immigrant communities about dementia and dementia care services:

*I think there must be a lot of open dialogue, open meetings, leaflets … People can come if you find some cases to present. Like some daughters, sons, uncles, sisters or brothers. If they say ‘I have a brother who has a problem’, I think it may open people to talk about dementia.*

According to this participant, the best way to raise awareness of dementia among minority ethnic groups is to give examples of people from the same ethnic group using dementia care services in Norway. This could facilitate the process of understanding dementia and help build trust in the Norwegian health care services.

## Discussion

The main aim of this qualitative study has been to identify barriers and facilitators in accessing and using dementia care services in Norway for people from minority ethnic groups. To gain a better understanding of the explored phenomena and a better overview of immigrants’ experiences, three groups of participants were included in the study: families from minority ethnic groups, representatives of health and care personnel, and representatives of immigrant communities. In light of the findings, we have chosen to use the theoretical perspective of intersectionality [37] to interpret some of the results. According to the theory of intersectionality, it is impossible to reduce human experience to single markers due to its rooting in social, historical and geographical contexts [38]. As an analytical tool, intersectionality allows researchers to explore the complexity of social life [39]. Thus, we look at the interconnectedness of various characteristics describing immigrants’ position in the host country, as this may influence the experiences of people from minority ethnic groups in accessing and using dementia care services in Norway.

The study indicates that there is a complex interplay of factors affecting access to and use of dementia care services in Norway for individuals from minority ethnic groups, and that cross-cultural differences cannot be explained by ethnicity alone.

In our study, we found that one of the main barriers to accessing dementia care services for people from minority ethnic groups in Norway were lack of knowledge and misconceptions about dementia, especially regarding symptoms. Initial symptoms of dementia, such as memory loss, were often treated as a normal part of the ageing process and did not prompt the participants to consult a doctor. These findings are in line with previous research from Norway [12] and from other countries [40–43]. According to our study, some individuals showing signs of dementia and their families from certain immigrant groups do not seek professional help because they believe the illness to be an act of God. This was related to dementia being understood as God’s will and, in result, the persons with dementia symptoms and their families do not seek professional help because they don’t want to act against God. According to a review of studies on ethnicity and pathways to care in dementia, minority ethnic groups give various non-neurological causal explanations of dementia, in addition to a belief that dementia is a normal part of the ageing process [42]. However, these studies do not necessarily look at perceptions and associated health seeking patterns in the light of socio-economic position, which may lead to an overemphasis on the influence of ethnicity [44]. A more recent quantitative Danish study on knowledge and perceptions of dementia and Alzheimer’s disease in minority ethnic groups in Copenhagen shows that higher education and degree of acculturation contributed to dementia knowledge [45]. The findings from our study indicate that many families from minority groups are unaware of the dementia care services available. Knowledge about the available services is a prerequisite for using them. For example, a quantitative study of Korean Americans’ attitudes towards community-based care services for dementia showed that those who were aware of the services available were more likely to have favourable attitudes towards using them [46].

As our study shows, in cases of patients with cognitive impairment, their lack of language skills hinders contact with health care institutions at every stage, from the diagnostic process to establishing contact with home care services, day centres and nursing homes. Studies from a variety of countries on various immigrant groups indicate that a lack of language skills may prevent immigrants from using health services [40, 47–49]. A review of articles on minority ethnic groups in dementia care shows that language ability was more important for the process of dementia assessment than minority ethnic group membership [50]. Using interpreters during the assessment only partially eliminates the language barrier. According to the literature, interpreters’ lack of experience in dementia assessment [31, 51, 52] and the lack of culturally and linguistically adapted assessment tools can complicate the whole process [31].

However, our study also highlights the importance of cultural background for accessing and using the health services. In particular, we found that caregiving regimes in the country of origin influence the way the families seek help and define their roles as caregivers. Hochschild, an American sociologist, distinguished between four cultural models of care/care ideals: warm traditional, cold modern, warm modern and cold postmodern [53]. The findings of our study provide examples of families coming from societies characterised by emotionally warm values of caregiving and familialistic caregiving regimes. However, we found that in addition to their country background, immigrant families’ caregiving ideals are shaped by many other factors, including socio-economic status, migration experiences and intergenerational solidarity.

Another important finding of our study is related to the children of people living with dementia who have to negotiate between their parents’ care expectations as reflected in the caregiving culture in their country of origin and Norwegian care ideals. Some of them, initiated into two different cultures, care traditions, moralities and values, seem to live on the margins of two cultures, like the “marginal man” described by Park [54]. They are characterised by a kind of transnational morality, trying to navigate between two different axionormative systems. These caregivers try to satisfy their parents’ care needs by providing care in the family, but at the same time they were ready to accept help provided by the state as long as their parents agreed. Our findings highlight the importance of levels of education and acculturation for immigrants’ attitudes towards family care and the care provided by institutions. Referring to intersectionality as our analytic tool, the findings show how various factors work together and position people differently in a particular social context [38]. The participants who were well educated and more socialised into Norwegian culture seemed to accept the Norwegian model of dementia care more than the first generation of immigrants. Our findings are in line with previous studies from Europe, US and Australia on migrants’ knowledge of dementia, and awareness and acceptability of services [45, 55, 56]. For example, and American study exploring dementia awareness among Korean Americans found that high levels of education and acculturation were associated with greater awareness of dementia care services and dementia [55].

Another factor that hinders accessing and using dementia care services is the perception of dementia as a taboo subject. According to our study, dementia can be perceived as something shameful. The stigma attached to people living with dementia and their families is one of the main reasons for dementia care deficits in many societies [57, 58]. The high level of stigma associated with dementia leads to a reluctance to acknowledge the symptoms, which in turn delays formal help-seeking and diagnosis [59, 60]. According to a British study among British Indian, African and Caribbean, and East and Central European minority ethnic groups, cultural stigma was reported as the main barrier to accessing dementia care services [40]. However, our study indicates the need to look beyond cultural factors to explain the existing stigma because it may be the result of an interplay of different factors defining the social status of individuals. A systematic review of research on stigma in the context of dementia showed that stigma was significantly correlated with factors such as educational level, age, gender and familiarity with someone with dementia [61]. Less stigma was reported among older people, females, those who knew someone living with dementia and those with higher levels of education [61].

The findings of our study point to the existence of many barriers in immigrants’ access to dementia care on the health care providers’ side, starting with general practitioners – the gatekeepers in the Norwegian health system – and ending with nursing homes. According to our study participants, some GPs perceived memory loss as a normal attribute of ageing and did not seem to have enough time for patients with special needs. There were examples of doctors putting off performing the necessary tests or not being aware of/familiar with culturally and linguistically adopted diagnostic tools; GP’s having the main responsibility of diagnosing dementia in Norway. In cases of insecurity about the diagnosis, there were examples of GP’s underestimating the symptoms, rather than referring patients to a specialist. These findings are in line with previous studies on barriers and facilitators in accessing dementia care by minority ethnic groups [12, 40, 42]. For example, a British study of perceptions of dementia and use of dementia care services among various minority ethnic groups found that they often had negative experiences of health care services, and of GPs in particular [40]. Some of them perceived GPs as too busy to provide care and not interested in their patients. A meta-synthesis of qualitative studies on barriers and facilitators in accessing dementia care by ethnic minority groups shows a lack of specialist dementia knowledge among GPs [28].

Our study highlights the importance of involving and supporting the families in all stages of the patient’s diagnostic process, treatment and care. Involving the families may facilitate the process of dementia diagnosis and help build trust between families from minority ethnic groups and health care providers. The relatives of patients with dementia often do not want to lose their role as caregivers, and our study shows that they expect health care providers to recognise them as an important source of information and support in the care provided. In general, our participants were not against using public dementia care services in Norway. However, they expressed a need for more culturally appropriate and flexible services, which is in line with a recent Norwegian study showing that immigrant families were willing to use dementia care services but wanted to do so in their own way [12]. Our findings suggest that Norwegian nursing homes and community-based home care are not prepared for patients from minority ethnic groups due to a lack of cultural sensitivity regarding food, religion, traditions and language. The findings are in line with other studies that highlight immigrants’ experiences of poor awareness of and cultural sensitivity towards different aspects of dementia care services [12, 40, 42]. Furthermore, the interviewed nurses in our study indicated that there was a gap in their education regarding knowledge about immigrant groups and their health and care needs. Then again, in the light of the theory of intersectionality, it might be misleading to focus on specific immigrant groups’ needs. Rather, one should consider how the interplay between culture, religion, age, gender, education/health literacy and language competencies constitutes specific needs – and how to adjust to these needs.

Thus, our findings highlight the need for continuous development of patient-centred dementia care. All the ethnic groups are characterised by internal heterogeneity in terms of social status and demographic characteristics. Thus, looking at patients only through the lens of ethnicity may result in stereotyping and inadequate assessment of their health situation. Social and economic inequalities are important causes of ethnic inequalities in access to health services. An Italian quantitative study of immigrants from low- and middle-income countries and high- income countries attending an Italian university memory clinic suggests that only wealthier and well-integrated immigrants who had established social networks and visited their GP regularly were referred to the clinic [62]. As exemplified in our findings, factors such as level of education and the social status of the family or its social network may influence immigrants’ knowledge about dementia and their awareness and use of dementia care services.

Our study identified some factors that facilitated immigrants’ access to dementia care services. However, we found fewer facilitators than barriers, which is in line with similar studies according to a meta-synthesis of previous research [28]. According to our findings, having a medical doctor in the family was the most important facilitator in accessing dementia care services. Medical doctors acted as cultural mediators between their parents and health care personnel. This facilitated communication and shortened the time needed for diagnosing patients, which was especially important in cases where GPs had very limited time for each patient. In addition, they used their position to encourage their parents to visit their GP and to convince the GP to extend diagnostics. Our findings clearly show how the intersection of various factors such and family dynamics, education and socio-economic status shape individuals’ experiences.

Another facilitator in accessing dementia care services for people from minority ethnic groups was related to the organisation of dementia care services. Some of our participants found that, despite the lack of systemic solutions, some health and care personnel were able to find effective strategies to facilitate access to and use of dementia care services for people from minority ethnic groups.

The findings imply that people from minority ethnic groups should be recognised as individuals with specific, complex needs, and not only as representatives of particular ethnic groups. The theory of intersectionality highlights the significance of social institutions in solving social problems [39] , p.16. Inclusive, individual-oriented health care policies seem especially important in cases of patients living with cognitive impairments, as these patients cannot simply adapt to or integrate into the system.

### Study strengths and limitations

The main strength of the study is its relevance, as both cases of dementia and diversity among those in need of services, will continue to increase. Another strength of this study is that it explored the perspectives of different actors in order to gain a broad overview of the challenges and facilitators in the access and use of dementia care by people from minority ethnic groups. The study sample included individuals from minority ethnic groups with family members experiencing dementia, key representatives of immigrant communities and representatives of health personnel working with people with dementia patients. This triangulation made us aware of barriers described in the same way across different groups of participants; thus validated as important factors to address. However, it also made us aware of barriers that was portrayed differently by the different participants, and that sometimes had its roots in misunderstandings or a lack of knowledge of the rationale behind treatment and care practises. The primary weakness is the small size of our geographically defined sample. We are aware that we might have not captured all the possible experiences, beliefs and opinions due to the complexity of the studied topic.

Our study clearly supports the previous studies on dementia in minority ethnic groups in Norway [12, 20, 31]. Based on rather consistent knowledge, it is now time for intervention studies aimed at facilitating access to dementia care services for people from minority ethnic groups.

## Conclusions

In order to provide equitable access to dementia care for people representing different linguistic, cultural and social backgrounds, it is necessary to identify the most important factors hindering and facilitating ethnic minorities’ access to dementia care services. The findings of this study indicate that there are several barriers related to both minority ethnic groups and health care providers. The study shows the importance of understanding the interplay of factors such as educational level, language competencies, cultural preferences, gender role expectations, level of acculturation in the host society and socio-economic status, in order to facilitate access to and use of dementia care services.

In order to ensure that people from minority ethnic groups receive appropriate dementia care, there is a need to develop inclusive policies that promote patient-centred approaches that do not overestimate the role of culture and ethnicity. Furthermore, there is a need for greater awareness of the symptoms of dementia and of the available public resources that can provide or complement the care provided by the family.

## Supplementary information

**Additional file 1.**

**Additional file 2.**

**Additional file 3.**

## Data Availability

The datasets (transcripts) generated and analysed during the current study are not publicly available due to risk of recognizing the participants. Transcripts are available from the corresponding author on reasonable request.

## Abbreviations

UN: United Nations
WHO: World Health Organization
AD: Alzheimer’s disease
GP: General practitioner
REK: Regional Committees for Medical and Health Research Ethics
NSD: Norwegian Centre for Research Data

## Footnotes

1 The concept of habitus was developed by Bourdieu. It refers to embodiment of cultural representations in human habits and routines. People’s behaviour and thinking are guided by socialised norms.
