# Supplementary material for: “It is always me against the Norwegian system.” barriers and facilitators in accessing and using dementia care by minority ethnic groups in Norway: a qualitative study
Source: BMC Health Serv Res. 2020 Oct 15;20:954. doi: 10.1186/s12913-020-05801-6 (PMC7565363; doi:10.1186/s12913-020-05801-6)
Supplement: Supplementary file 1 — Additional file 1. [file 12913_2020_5801_MOESM1_ESM.docx]

**Interview with families**

**Warming up questions.**

**Dementia – related issues: the level of memory impairment, the need for care, behavior, daily functioning.**

Could you describe your family? Who in your family suffers from dementia?

How old is he/she? What is her/his education background? What is her profession?

How could you describe the level of her/his memory impairment?

Does she/he need help in daily functioning? What kind of help? How is his/her daily functioning?

How could you describe her/his behavior?

**Experiences related to the onset and development of the disease (the first symptoms, diagnosis)**

I would like to ask you about the beginning of dementia/memory problems in your family. How did you realize that something wrong was happening with your mother/father?

What were the first symptoms of the disease?

Do you remember your reactions? How did you feel? How did the other family members react?

**Understanding of dementia**

What did you know about dementia before it appeared in your family? Do you know more now? What are the sources of your knowledge on dementia?

How is dementia perceived in Norwegian society? What do people in your country think about dementia?

What do you think about causes of dementia? Why do you think your family member got ill? Is it possible to prevent dementia/memory impairment?

**Caring in the family**

Does your family participate in caregiving? How? Why?

How could you define a good care?

How does your family/you cope with care giving? How was it at the beginning and how is it now? What helps you when it gets difficult? What do you do to comfort yourself?

**Available social support**

Is there anyone who can help you to care for your mother/father? Who?

How do they help you? What do you think about their help?

**Seeking of formal care, experiences of care provided by health services**

Why did your family come in touch with health care institution? When? How long after they observed the first symptoms? Who contacted the health care institution?

What prevents/prevented you from seeking help?

How did you find out about the available support? Was is difficult to get professional help? Do you collaborate with the institution? How could you describe the collaboration with the institution? What works well? What should be changed or improved?

**Family care versus institutional care**

In your opinion, what is the best for the person with dementia, family care or the institutional care? What is the best solution for the family?

Did you take care of your mother/father at home? Do you still provide care while your relative is in the institution? What kind of care?

How was it the first time to meet your relative in the institution? Do you remember how you felt at the beginning?

**The current situation of the family**

How does your family manage the situation? How do you feel?

Do you need support? From whom? What would make your situation easier?

Do you need information on dementia? What kind of information? How would you like to get it?

**Information about the family members**

socio-demographic characteristics: gender, age, education, profession, length of stay in Norway, self-assessment of health, financial situation.
