# Supplementary material for: “It is always me against the Norwegian system.” barriers and facilitators in accessing and using dementia care by minority ethnic groups in Norway: a qualitative study
Source: BMC Health Serv Res. 2020 Oct 15;20:954. doi: 10.1186/s12913-020-05801-6 (PMC7565363; doi:10.1186/s12913-020-05801-6)
Supplement: Supplementary file 2 — Additional file 2. [file 12913_2020_5801_MOESM2_ESM.docx]

**Interview with key representatives of migrant communities**

**A process of ageing**

What are most important characteristics of ageing in your opinion?

Does aging affect emotional wellbeing?

**The role of old people in the society**

What is the role of elderly people in xxx (Pakistan, Somalia…)?

What is the role of elderly people in xxx (Pakistani, Somali…) families in Norway?

What is the role of elderly people in Norway?

**Understanding of dementia**

What do you know about dementia?

How is dementia perceived in Norwegian society? What do people in your country think about dementia?

What do you think about causes of dementia?

Is it possible to prevent dementia/memory impairment?

**Care for the elderly in the family**

How would you describe the family care for the elderly in xxx (Pakistan, Somalia…) and in Norway? Are there any differences?

Do xxx (Pakistani, Somali…) families feel obliged to provide care for the elderly? Who in the family is the main caregiver?

How could you define a good care? What does it mean?

**The role of health care services in taking care for the elderly**

Could you describe the institutional care for the elderly in xxx (Pakistan, Somalia…)?

What is the attitude of xxx (Pakistanis, Somalis…) towards the institutional care/health care services? Why?

What do you think, what is the best for the person with dementia, family care or the institutional care? What is the best solution for the family?

What would be the best support for xxx families with a person suffering from memory impairment?

What can prevent xxx (Pakistani, Somali…) families with dementia problem from seeking help in Norway?

What do xxx (Pakistani, Somali…) families do when a family member starts having problems with memory?

**Socio-demographic characteristics**: gender, age, education, profession, length of stay in Norway, self-assessment of health, financial situation.
