# Supplementary material for: “It is always me against the Norwegian system.” barriers and facilitators in accessing and using dementia care by minority ethnic groups in Norway: a qualitative study
Source: BMC Health Serv Res. 2020 Oct 15;20:954. doi: 10.1186/s12913-020-05801-6 (PMC7565363; doi:10.1186/s12913-020-05801-6)
Supplement: Supplementary file 3 — Additional file 3. [file 12913_2020_5801_MOESM3_ESM.docx]

**Interview with representatives of health and care personnel**

**Experiences with patients from minority ethnic groups with memory impairment (differences between Norwegian patients and patients from minority ethnic groups)**

Do you work with such patients? From which minority groups?

What are your experiences?

Did you experience any differences between Norwegian patients and patients from minority ethnic groups? Can you say something about it?

**Experiences with the families (**collaboration with the families, their understanding of dementia, family’s role in caring for the person with dementia, seeking help**)**

**Which services work well / which services don’t work in case of the patients from minority ethnic groups and their families (**examples, barriers and facilitators in access to and use of the services by the members of ethnic minority groups**)**

**Challenges for health and care personnel and family caregivers related to caring for patients from minority ethnic groups**

What are the challenges for nursing homes’ employees related to having patients from minority ethnic groups?

What are the challenges for the families of those patients?

What could be done to overcome those challenges (for the employees and the families)?

**How to improve minority ethnic groups’ access to healthcare services** **in Norway**
